# Entrepreneurship Under Fire: Psychological Distress During Armed Conflict from a Public Health Perspective

**DOI:** 10.3390/ijerph22121866

**Published:** 2025-12-15

**Authors:** Sharon Hadad, Ohad Shaked

**Affiliations:** 1 Department of Economics, Sapir Academic College, Sderot 7916500, Israel; 2 Department of Technological Marketing, Sapir Academic College, Sderot 7916500, Israel; shakohad@mail.sapir.ac.il

**Keywords:** terrorism, armed conflict, distress, small business, entrepreneurship, community support, psychological distress, resilience factors, government support

## Abstract

On 7 October 2023, Israel experienced a large-scale attack, initiating the Iron Swords War (also known internationally as the 2023 Israel–Hamas War). This protracted armed conflict profoundly disrupted social and economic life in Israel and the region. This study investigates the psychological distress of small business owners in the aftermath of this terrorist assault and during the ensuing conflict. Drawing on a nationwide survey of 363 entrepreneurs, we applied a two-stage higher-order PLS-SEM model to examine how economic stressors, psychological and institutional resources, and demographic factors shaped distress. The findings reveal that uncertainty and revenue loss intensified distress, while resilience, hope, and trust in government operated as protective resources, with notable gender differences also observed. Beyond its economic and psychological relevance, the study situates entrepreneurial distress within a broader public health perspective, viewing the mental health and well-being of small business owners as integral to community resilience, social stability, and national recovery during crises. By framing entrepreneurial distress and resilience as key determinants of population mental health and collective well-being, this research underscores how supporting entrepreneurs contributes to wider health promotion and psychosocial recovery efforts. Overall, the study offers a novel multidimensional empirical analysis of entrepreneurial distress during armed conflict, underscoring the psychological mechanisms through which terrorism and its aftermath affect small business owners, and highlighting the need for resilience-building and institutional support to mitigate mental health burdens.

## 1. Introduction

Small businesses are the cornerstone of global economies, with micro, small, and medium enterprises (MSMEs) accounting for about 95% of all firms worldwide and generating approximately 60% of global employment [1]. Despite their pivotal role in job creation and growth, these enterprises are particularly vulnerable to external shocks, such as pandemics, natural disasters, or violent conflicts. Their susceptibility arises not only from limited financial and managerial resources, a lack of formal crisis preparedness, and a reactive approach to risk management [2], but also from the unique overlap between the entrepreneur and the business itself. In MSMEs, decision-making is largely owner-driven, meaning that the personal well-being, resilience, and psychological state of the entrepreneur directly shape the firm’s capacity to withstand and adapt to crises [3].

Such vulnerabilities become especially evident when abrupt changes in market demand or supply chain disruptions threaten survival [4]. The steep decline in small business activity during the COVID-19 pandemic [5] exemplifies how crises can quickly escalate into existential threats, an issue no less acute in contexts of war and terrorism, where uncertainty and insecurity compound economic challenges. At the same time, research on entrepreneurial resilience shows that psychological and social resources can buffer the impact of crises and support continued business activity under uncertainty [6,7,8,9].

Building on this perspective, evidence from conflict-affected and post-conflict economies shows that MSMEs are widely recognised as key engines of economic recovery, where entrepreneurship supports job creation, local growth, and social stability [10,11,12]. Yet, the literature indicates that the role and impact of entrepreneurship in such settings remains underexplored, particularly with respect to how entrepreneurs themselves navigate conflict-related risks, constraints, and psychological strain. By focusing on the mental health and resilience of small business owners during an ongoing armed conflict, this study directly addresses this gap and underscores the public health relevance of entrepreneurial well-being for economic recovery.

Israel provides a compelling context for studying entrepreneurial resilience due to its history of managing periods of armed conflict, security threats, and national emergencies. MSMEs dominate the Israeli economy, accounting for roughly 99.5% of all firms and about 55% of the business sector’s output [13]. These businesses are vital for employment and economic growth, yet they are highly vulnerable to external shocks, particularly in crisis-prone sectors such as retail, hospitality, and tourism. Importantly, the Iron Swords War struck just as the business sector was beginning to recover from the severe disruptions of the COVID-19 pandemic, amplifying the strain on entrepreneurs and further underscoring the fragility of MSMEs in Israel’s volatile environment.

The attacks in Israel on 7 October 2023 resulted in significant casualties and the abduction of civilians [14]. For a short period, Israeli territory was seized, creating a sense of vulnerability. The scale of the event has had a profound national impact, extending beyond the immediate casualties. Many households in Israel were affected, whether through bereavement or the emotional impact of uncertainty and fear [15]. The subsequent Iron Swords War has become a protracted conflict, reshaping daily life and national priorities. In addition to the human and emotional toll, the conflict generated social and economic disruptions. Families faced prolonged separation due to reserve enlistments, producing strain and challenges [16].

On a macroeconomic scale, the war severely disrupted labour markets and supply chains, leading to the closure of approximately 75,000 small and medium-sized enterprises, 80% of which were micro-enterprises [17]. The economic damage, estimated at more than NIS 350 billion [18], has strained Israel’s GDP and revealed the fragility of its small business sector. Macroeconomic indicators reflect the scale of the shock: in the last quarter of 2023, GDP contracted at an annualized rate of 21% due to declines in private consumption, exports, and business investment, while overall growth for 2024 was limited to just 0.7% compared to more than the 3% recorded in previous years. The budget deficit increased to 6.8% of the GDP in 2024, the highest since the COVID-19 pandemic, while the debt-to-GDP ratio climbed from around 60% before the war to nearly 70% in 2025. Tourism collapsed, with arrivals plunging by about 80% in Q4 2023 compared to the prior year, and remained deeply depressed throughout 2024 [19]. Survey data indicated that 56% of business owners reported steep declines in activity, particularly in sectors dependent on local and regional demand [20]. These circumstances highlight the significant impact of the Iron Swords War, encompassing humanitarian, economic, psychological, and entrepreneurial dimensions. From a public health perspective, the psychological and economic disruptions experienced by entrepreneurs extend beyond individual hardship and affect employment stability, local service provision, and community functioning. Mental health problems such as anxiety, stress, and burnout among small business owners can cascade to employees, customers, and family members, whereas resilience and supportive environments may help to buffer these ripple effects. Understanding how psychological distress and resilience manifest among entrepreneurs during crises, therefore, provides insight into the behavioural and social determinants of health that are central to contemporary public health policy [21,22,23,24]. Accordingly, the objective of this study is to investigate how economic, psychological, and institutional factors jointly shape psychological distress among small business owners during the Iron Swords War, and to identify the mechanisms through which these effects operate.

## 2. Theoretical Framework

### 2.1. Introduction

Our theoretical framework is informed by public mental health and social determinants of health perspectives. Research on disasters and mass trauma conceptualises psychological distress as a non-specific mental health response that emerges when individuals are exposed to severe stressors and experience losses of key resources, uncertainty, and diminished control over their lives [25,26,27,28]. In the context of small business ownership, these stressors include financial losses, threats to business continuity, and insecurity regarding the future. At the same time, personal and contextual resources such as resilience, hope, and trust in institutions can buffer the impact of these exposures. This framework motivates our focus on economic, psychological, institutional, and demographic determinants of distress in the empirical model.

### 2.2. Entrepreneurial Psychological Distress in Crises

Crises such as wars, terrorism, natural disasters, and pandemics generate not only economic disruption but also substantial psychological strain. Psychological distress, typically manifesting as anxiety, depression, and stress, reflects both individual responses and the broader socio-economic pressures that accompany such shocks [29,30]. Research on events like 9/11, natural disasters, and the COVID-19 pandemic shows increased psychological morbidity, diminished well-being, and impaired decision-making during times of acute uncertainty [26,31,32].

MSME entrepreneurs are especially susceptible, as their personal and professional domains are closely intertwined. Disruptions that threaten business continuity simultaneously undermine family stability and personal security, thereby intensifying stress [33]. In Israel, where exposure to wars and terrorism is recurrent, such vulnerabilities are magnified. Beyond individual health, distress also has economic repercussions, limiting entrepreneurs’ ability to make effective decisions and jeopardizing business resilience. In addition to this broader crisis literature, micro-level studies in fragile and conflict-affected settings show that small business owners often use business activity as a coping mechanism and as a source of socio-economic resilience under protracted violence [34,35]. Emerging evidence further suggests that psychosocial interventions that improve the mental well-being of SME owners can enhance productivity and livelihoods [36]. Together, these findings indicate that the resilience of small business owners is not only an economic issue but also a public health concern in conflict-affected contexts, thereby motivating our focus on entrepreneurial distress during the Iron Swords War.

### 2.3. Risk Factors Shaping Distress

Several factors have been identified in the literature as key drivers of psychological distress among entrepreneurs during crises. Economic stressors are often at the forefront, as revenue losses and the looming risk of business closure generate severe strain. Financial pressure not only undermines business continuity but also erodes entrepreneurs’ sense of control, thereby amplifying distress [37].

Uncertainty has been repeatedly highlighted as one of the most powerful predictors of psychological strain. Entrepreneurs facing crises often contend with unclear time horizons, unpredictable customer behaviour, and ambiguity regarding government support. Such uncertainty exemplifies Knightian uncertainty, a type of immeasurable uncertainty where probabilities cannot be assigned, and decision-making relies heavily on entrepreneurial judgment [38]. This magnifies the sense of vulnerability and helplessness, restricting the capacity to plan ahead or invest in recovery.

Another important factor is trust in government and institutions. When entrepreneurs perceive institutional responses as insufficient or unreliable, their distress levels increase, as diminished trust translates into a heightened sense of abandonment and lack of support [38]. In conflict settings, such trust is often intertwined with perceptions of whether the war is being managed responsibly and pursued for legitimate reasons, so lower confidence in the government’s handling of the war and the emergency context can exacerbate psychological distress among business owners.

Finally, demographic factors play a significant role. Gender differences are particularly notable, with studies consistently showing that women entrepreneurs report higher levels of distress compared to men. These differences may stem from the dual burden of professional and family responsibilities, as well as broader structural inequalities that heighten vulnerability during crises [39,40]. In Israel, during the Iron Swords War, the burden on women entrepreneurs was further amplified as many men were conscripted for prolonged and repeated reserve duty. The absence of their partners from household tasks and childcare, coupled with the constant concern for their safety on the battlefield, exacerbated women’s stress and intensified the challenges of balancing business continuity with family demands [41].

Together, these risk factors provide a multidimensional view of how distress emerges and intensifies in entrepreneurial contexts, underscoring the need to consider both structural conditions and individual characteristics in understanding entrepreneurial resilience.

### 2.4. Protective Psychological and Social Resources

While crises expose entrepreneurs to significant distress, the literature also highlights protective factors that can mitigate these effects and foster resilience. Resilience, broadly defined as the capacity to adapt under pressure, plays a central role in buffering the psychological impact of crises and sustaining business activity under uncertainty [6,7]. In the literature, resilience is often conceptualized as a construct that integrates both personal and social dimensions [42]. Two particularly salient components are self-efficacy, i.e., the individual’s belief in their ability to effectively manage challenges [6,43], and social and community support, i.e., the perception of available emotional, moral, and practical assistance from family, peers, and institutions [8,9]. Self-efficacy strengthens confidence and persistence, while social support provides a relational safety net that mitigates isolation and strain. Together, these resources reinforce resilience and reduce psychological vulnerability during crises. This perspective accords with conservation of resources (COR) theory, which explains psychological stress as a response to the loss or threatened loss of valued resources and views resilience as the ability to mobilise clusters of personal and social resources, or “resource caravans”, rather than isolated attributes [44,45]. In line with this view, we conceptualise entrepreneurial resilience in this study as a configuration of resilience-related resources, in particular self-efficacy and perceived social support, that jointly buffer distress in wartime conditions.

Another protective mechanism is hope, which represents a positive cognitive–emotional orientation toward the future. Hope fosters the perception of opportunities even amidst uncertainty and reduces feelings of helplessness. For entrepreneurs, hope supports the development of adaptive strategies, encourages innovation, and promotes endurance when resources appear scarce [7,46].

Taken together, these protective resources underscore the multidimensional nature of entrepreneurial coping strategies. They highlight that psychological strengths and social ties are not merely individual attributes but vital assets that can safeguard entrepreneurs’ well-being and enhance their capacity to endure and adapt to crises.

## 3. Research Gap and Study Contribution

The psychological dimensions of entrepreneurship, including resilience, self-efficacy, and hope, have been the subject of extensive research across diverse contexts. These studies consistently demonstrate the importance of psychological and social resources in helping entrepreneurs withstand economic shocks and uncertainty. Yet, there is limited research examining these dynamics in situations of war or terrorist attacks, where the intensity of threat, disruption, and insecurity is qualitatively different from other crises. In such circumstances, small business owners are simultaneously exposed to financial losses, personal risk, and institutional fragility, which may fundamentally alter how protective mechanisms operate and how distress is experienced.

The present study bridges this gap by providing one of the first empirical, multidimensional analyses of psychological distress among small business owners during a large-scale, protracted armed conflict. Grounded in the literature on entrepreneurial coping, crisis management, and psychological resources, this study examines how economic shocks (i.e., revenue loss) interact with psychological resources (resilience, support, and hope), institutional factors (trust in government), and demographic variables (gender) to shape distress levels. A key theoretical innovation of this study lies in its hierarchical modeling of entrepreneurial resilience as a higher-order construct composed of self-efficacy and social support, an approach seldom undertaken in applied resilience research. Moreover, by explicitly testing direct and indirect pathways, from economic stressors through uncertainty and psychological resources to distress, this research advances beyond prior studies on entrepreneurship in crisis contexts, which have often focused primarily on linear or isolated associations.

The central research question guiding this study is as follows: How do economic, psychological, institutional, and demographic factors jointly influence psychological distress among small business owners during armed conflict, and through what mechanisms are these effects channelled?

To address this question, we applied a two-stage higher-order PLS-SEM model. This approach is novel in the context of entrepreneurial resilience during armed conflict, as it enables the simultaneous estimation of latent constructs and hierarchical relationships, while capturing both direct and mediated pathways that are difficult to assess with simpler methods such as correlations or OLS regression. Operationally, the economic factors included perceived revenue loss and uncertainty about the crisis, the psychological factors included resilience (modelled as a higher-order construct composed of self-efficacy and support) and hope, and the institutional factor was trust in government institutions. In addition, gender was included as the demographic control variable.

## 4. Methods

This section describes the study design in four parts: the sample, the measurement of constructs, preliminary analyses (correlations, *t*-tests, regressions), and the partial least squares structural equation model (PLS-SEM) with a two-stage higher-order construct (HOC) approach.

### 4.1. Sample

The data for this study were derived from a nationwide survey conducted during the initial months of the 2023 Iron Swords War (Ethical approval for this study was granted by the Ethics Committee of Sapir Academic College (Approval No. 27072025)). The sample consisted of 363 small business owners across Israel. Among the respondents, 72.5% were women, and 27.5% were men. Such overrepresentation of women among survey respondents is consistent with prior evidence that women tend to participate more often than men in survey research [47,48]. In addition, in the present wartime context, many men were serving in reserve military duty at the time of the survey and were therefore less available to participate, resulting in a higher proportion of female respondents.

In terms of geographic location, 41.3% resided in peripheral regions, while 48.8% were located in central areas. Participants operated in a wide range of sectors, including services, tourism, finance, real estate, food and hospitality, leisure and creative industries, professional consulting, education, and health, with the full sectoral distribution presented in Table A1.

In addition to demographic background variables, the survey included validated measures of psychological distress, as well as trust in government institutions, resilience, self-efficacy, levels of hope, perceived uncertainty about the crisis, and reported revenue loss. Gender was treated as a control variable due to the uneven distribution in the sample, reflecting the profile of survey respondents as obtained by the survey firm.

This multidimensional dataset provides a comprehensive picture of the experiences of entrepreneurs during a national emergency, enabling the identification of key psychological, economic, and demographic factors associated with distress in times of crisis.

### 4.2. Constructs and Measures

Most constructs were measured using items adapted from validated scales where available, while some were developed ad hoc for the current study (see Appendix A for the complete list of items, codes, and sources). Each construct underwent reliability and validity testing, with detailed indices (Cronbach’s α, Composite Reliability, AVE, and discriminant validity) reported in Section 5 (Measurement Model Results) and in Appendix B.

- Psychological Distress was assessed using three items capturing anxiety, stress, and depressive symptoms related to the situation of war. Items were adapted from the Kessler Psychological Distress Scale [25] and the DASS-21 [49]. Example item: *“I feel Stress about the current situation.”*
- Hope was measured using three items reflecting expectations for a positive business future despite the war, adapted from Snyder’s Hope Scale [50] and the Herth Hope Index [51]. Example item: *“I hope that after the war, the future of my business will be good.”*
- Trust in Government institutions was measured using four items adapted from previous research on political trust [52]. The items assessed confidence in governmental institutions to support businesses during and after the crisis. Example item: *“I trust the state and governmental institutions to provide the necessary support to businesses in the future.”*
- Resilience was measured with eight items adapted from the Connor–Davidson Resilience Scale [53]. These items reflect the perceived capacity to adapt positively to adversity, to transform challenges into opportunities, and to persevere under stressful conditions. Example item: *“I believe that I can grow in positive ways by coping with difficult situations.”*
- Self-Efficacy was measured with six items adapted from the General Self-Efficacy Scale [54]. These items capture confidence in one’s ability to manage business challenges, implement new ideas, and take initiative in problem-solving. Example item: *“I always try to find a way to overcome obstacles; I believe in my ability to overcome challenges.”*
- Uncertainty was operationalized as the perceived unpredictability regarding the course of the war and its implications for business. It was measured using three context-specific items developed for this study, reflecting uncertainty about governmental support, customer behaviour, and the continuation of the war.
- Support was measured with six items developed ad hoc for this study, capturing perceived family and community support during the war. The items covered the instrumental, emotional, and social dimensions of support. Example item: *“My family and community provide me with connections that help me advance my business.”*
- Revenue Loss was measured on a six-point self-report scale ranging from “growth in revenue” (1) to “business closure or a 100% decline in revenue” (6). The average reported level of financial damage was moderate (M = 3.27, SD = 1.29).
- Gender was included as a control variable (coded 0 = male, 1 = female), given the skewed distribution in the sample.

### 4.3. Data Analysis

As an initial step, we examined bivariate associations between the study variables to establish a baseline understanding of their interrelations. These preliminary analyses provided a first indication of which psychological, economic, and demographic factors were most closely linked to small business owners’ distress during the Iron Swords War. We then estimated an ordinary least squares (OLS) regression model to account for these factors simultaneously and to identify their unique contributions before proceeding to the more comprehensive PLS-SEM analysis.

#### 4.3.1. Bivariate Correlations

The strongest correlate of distress was perceived uncertainty regarding the future of the crisis (r = 0.456, *p* < 0.01), highlighting the psychological burden of not knowing how long the conflict would persist. A smaller but statistically significant positive correlation was also found between revenue loss and distress (r = 0.113, *p* = 0.03), indicating that economic hardship contributes to emotional strain.

Conversely, resilience was negatively correlated with distress (r = −0.127, *p* = 0.01), suggesting that psychological resources may serve as protective factors during crisis situations. Additionally, a strong negative correlation was also found between trust in government institutions and distress (r = −0.31, *p* = 0.01), implying that decreased institutional trust may amplify psychological burden.

Hope also emerged as a significant variable: entrepreneurs with higher levels of hope reported significantly lower levels of distress (r = −0.198, *p* < 0.01).

In addition, independent-sample *t*-tests revealed that women reported significantly higher levels of distress than men (T (361) = −7.05, *p* = 0.01).

Taken together, these findings suggest that psychological distress during the war was shaped by a combination of psychological factors (e.g., hope, resilience, uncertainty, and trust), economic conditions (e.g., revenue decline), and sociodemographic dimensions such as gender. These bivariate results are summarized in Table 1.

#### 4.3.2. OLS Regression

In the second stage of analysis, we estimated an ordinary least squares (OLS) regression to predict levels of distress among small business owners during the Iron Swords War. The model yielded a significant overall fit, with F (6, 356) = 29.95 and *p* < 0.001, and explained approximately 33.5% of the variance in distress levels (R^2^ = 0.335, adjusted R^2^ = 0.324, N = 363). The regression results are presented in Table 2.

A noteworthy pattern emerged when moving from bivariate correlations to the multivariate OLS model. Although revenue loss and resilience correlated with distress in the expected directions, their regression coefficients were not significant once uncertainty, hope, trust in government, and gender were entered. To better understand these results and to model the latent constructs with their measurement properties, we proceeded to a PLS-SEM specification using a two-stage higher-order construct approach. This framework allows us to estimate direct and indirect effects simultaneously, capture the hierarchical structure within psychological resources, and evaluate theoretically hypothesized indirect pathways among economic stressors and psychological resources.

### 4.4. The PLS-SEM Model

We use a partial least squares structural equation model (PLS-SEM), which estimates latent variable scores and structural relations by maximizing explained variance. While OLS regressions provided initial insights, they could not account for the measurement of latent constructs. PLS-SEM, by contrast, is particularly appropriate given the need to model hierarchical constructs such as resilience. This approach enables us to simultaneously estimate direct and indirect effects and to evaluate theoretically specified pathways linking economic stressors, psychological resources, and distress.

**Figure 1:**
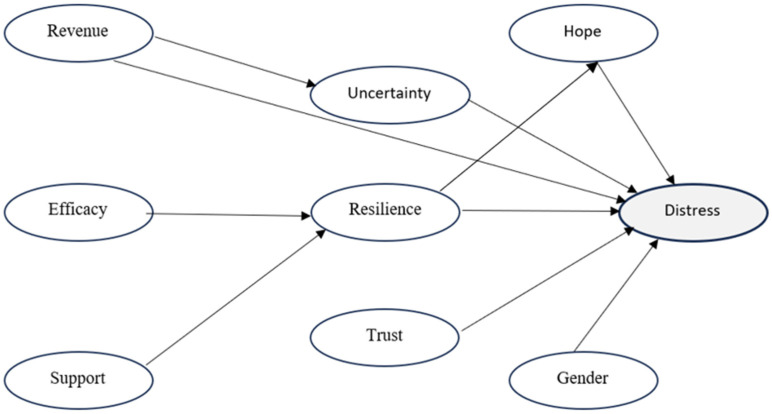
PLS-SEM specification with a two-stage higher-order resilience construct. Note: “Efficacy” denotes self-efficacy, “Revenue” denotes revenue loss, and “Trust” denotes trust in government.

Our PLS-SEM model uses a two-stage higher-order construct approach. All constructs were modelled reflectively (i.e., the observed items are taken as expressions of the underlying latent construct). In Stage 1, the lower-order resources’ self-efficacy and support were estimated from their observed indicators. In Stage 2, these components formed the higher-order resilience (HOC). The structural model specifies paths from revenue loss to uncertainty, from uncertainty to distress, from resilience (HOC) to hope, and from hope to distress, together with direct paths from revenue loss and resilience (HOC) to distress. Trust in government was specified as an additional explanatory variable, while gender was included as a control. Estimation was conducted in SmartPLS 4 with bootstrapping for inference. Convergent validity and reliability were assessed using indicator loadings, Cronbach’s alpha, composite reliability, and AVE, and discriminant validity was examined using the Fornell–Larcker criterion and HTMT. We report standardized path coefficients (β), two-tailed *p* values, and R^2^ for endogenous constructs. The conceptual specification is shown in Figure 1.

## 5. Model Results

### 5.1. Stage 1: Measurement Model Results

The analysis of the measurement model allows for investigating the relationship between the observed or measured variables and a latent variable [55]. Each latent construct was assessed using multiple survey items. The full model results are presented in Appendix B.

#### 5.1.1. Convergent Validity and Reliability

To evaluate the adequacy of the measurement model, we first examined the convergent validity and reliability. Indicator loadings were generally strong on their intended constructs (resilience λ = 0.686–0.831; self-efficacy = 0.746–0.852; support = 0.660–0.849; hope = 0.797–0.850; trust = 0.707–0.874; uncertainty = 0.693–0.857; distress = 0.818–0.935). Most constructs met the recommended criteria for reliability and convergent validity. Cronbach’s alpha ranged from 0.624 (uncertainty) to 0.886 (resilience), with composite reliability (CR) between 0.872 and 0.927 and AVE ranging from 0.559 to 0.810 across constructs. Although Cronbach’s α for the uncertainty construct was below the conventional 0.70 threshold (α = 0.624), the rho_A coefficient (0.68) approached the acceptable range, and both the composite reliability (0.882) and AVE (0.653) exceeded recommended cut-offs. This pattern is consistent with the view that rho_A provides a stronger estimate than α, particularly for short, newly developed scales [55]. Hence, we retained the construct in the model (see Table A2 in Appendix B).

#### 5.1.2. Discriminant Validity

This test evaluates whether each latent construct is empirically distinct from the others. In the two-stage model, discriminant validity is assessed only among the lower-order components. Specifically, the Fornell–Larcker criterion was met, and the HTMT values were below the recommended threshold (Self-Efficacy vs. Support HTMT = 0.447). Discriminant validity for the higher-order resilience construct vis-à-vis other constructs (hope, uncertainty, trust, and distress) is assessed in Stage 2 of the analysis.

Overall, the Stage 1 measurement model demonstrated adequate reliability and convergent validity across constructs and acceptable discriminant validity among the lower-order components. These results justified treating the indicators as reflective and using the latent scores as reliable inputs to the Stage 2 PLS-SEM structural analysis.

In Stage 2, the latent scores of the lower-order components (self-efficacy and support) were used to form the higher-order resilience construct. These scores then served as inputs for estimating the structural model. Multicollinearity among predictors was inspected with inner VIF and found to be below the recommended cut-offs, and inference relied on bootstrapping. We report standardized path coefficients, two-tailed *p* values, confidence intervals, and R^2^ for the endogenous constructs. Indirect effects were tested for the following paths: revenue loss → uncertainty → distress, and resilience → hope → distress.

### 5.2. Stage 2: Structural Model Results

The structural model yielded several noteworthy findings, based on the path coefficients reported in Table A5, Table A6 and Table A7 (Appendix B). First, gender was significantly associated with distress, with women reporting higher levels of distress than men (β = 0.701, *p* < 0.001). However, this result should be interpreted with caution, as the wartime data collection led to a substantial gender imbalance in the sample, limiting the extent to which this finding reflects true population-level gender differences. Among the psychological resources, hope reduced distress significantly (β = –0.151, *p* = 0.001), while resilience did not exert a direct effect (β = –0.024, *p* = 0.62). Instead, resilience influenced distress indirectly through hope (β = –0.057, *p* = 0.002). When both direct and indirect effects are considered, the total effect of resilience on distress is negative but only marginally significant (β = –0.081, *p* = 0.072), suggesting that resilience alleviates distress mainly through its positive relationship with hope rather than by a strong direct impact. Trust in government also acted as a protective factor, showing a negative relationship with distress (β = –0.158, *p* = 0.002). By contrast, uncertainty about the future of the crisis increased distress (β = 0.358, *p* < 0.001), and revenue loss affected distress only indirectly through uncertainty (β = 0.083, *p* < 0.001), highlighting the psychological rather than purely financial channel through which economic hardship translated into emotional strain. Together, the model explains 36%, a substantial proportion, of the variance in distress (R^2^ = 0.361) (Table A7 in Appendix B). For a comprehensive overview of all direct, indirect, and total effects in the structural model, see Table A5, Table A6, Table A7 and Table A8 in Appendix B.

Collinearity assessment (inner VIF values) indicated that all predictors of distress had VIF values between 1.02 and 1.22, well below the conservative cut-off of 5. Thus, multicollinearity was not a concern (Table A9 in Appendix B).

Discriminant validity was also assessed using the heterotrait–monotrait (HTMT) ratio of correlations. All HTMT values were well below the conservative threshold of 0.85, thereby confirming satisfactory discriminant validity among the higher-order constructs in the structural model (Table A10 in Appendix B).

Overall, the model reveals that the impact of revenue loss on distress operates primarily through heightened uncertainty, while resilience alleviates distress mainly via its positive relationship with hope. These findings underscore the psychological mechanisms through which economic and personal resources shape entrepreneurs’ distress during crisis.

## 6. Discussion

With this study, we set out to explore how economic, psychological, and institutional factors jointly shape small business owners’ psychological distress during an armed conflict. Three main insights emerged in our results.

First, uncertainty is a key proximal driver of distress in this context. Although revenue loss correlated positively with distress in bivariate analysis, its direct effect was not significant in the multivariate PLS-SEM once uncertainty was included; instead, revenue loss affected distress indirectly via heightened uncertainty. We interpret this as evidence that, under conditions of war, the inability to anticipate the duration and consequences of the crisis becomes the dominant channel through which economic harm translates into psychological burden. This interpretation is consistent with judgment-based views of entrepreneurship under Knightian uncertainty and with the notion that institutional and political instability can amplify uncertainty, impinging directly on entrepreneurial decision-making. This also aligns with prior research emphasizing the disruptive effects of Knightian uncertainty on entrepreneurial judgment [38]. Our study extends this line of inquiry by revealing its psychological burden and dominance in a wartime entrepreneurial context. Although this pattern is consistent with long-standing findings in cognitive psychology, our contribution is to demonstrate that the same uncertainty-distress mechanism also operates among small business owners who manage firms under wartime conditions, and remains dominant even after accounting for financial loss, psychological resources, and institutional trust. In the full structural model, this pattern is reflected in the fact that most of the explained variance in distress is accounted for by uncertainty about the crisis and, within the limits of the present non-representative gender distribution, by gender, whereas resilience, hope, and institutional trust have smaller direct effects and appear to operate mainly through indirect pathways.

Nonetheless, this pattern does not preclude a significant direct link between revenue loss (or other financial indicators) and distress in other settings. In peacetime recessions, sudden negative demand shocks and financial strain may be sufficiently acute to elevate distress. Our results show that in an ongoing armed conflict, perceived uncertainty appears to overshadow the direct financial channel.

Second, psychological resources protect well-being, but mainly through hope. In the wartime context, resilience did not reduce distress directly but exerted its protective effect indirectly through hope, suggesting that resilient entrepreneurs cope better primarily because they sustain positive future-oriented expectations. This supports prior research that has conceptualized resilience as a multidimensional construct encompassing self-efficacy and social support [6,7,42] and research identifying hope as a critical psychological resource that fosters adaptive coping and persistence under adversity [7,46]. Our findings refine this literature by positioning hope not merely as a correlate of resilience, but as a central mechanism through which resilience exerts its protective effect during war. Third, Trust in government also buffered distress, complementing prior accounts which emphasize how diminished trust can heighten entrepreneurs’ sense of strain [38]. Our findings extend this perspective by showing that, under wartime conditions, institutional trust functions as a protective resource that reduces distress, consistent with frameworks highlighting the role of institutional clarity and credibility in dampening uncertainty burdens.

### Public Health Relevance and Implications

From a broader public health standpoint, these three insights underscore that the psychological resilience and well-being of entrepreneurs are not only private concerns but critical components of community mental health and social stability. Small business owners are often central figures in local economies, and their capacity to function effectively during crises influences employment, income distribution, and family well-being, factors directly tied to the social determinants of health [21,22]. Thus, interventions that strengthen entrepreneurial resilience, institutional trust, and hope have the potential to yield population-level benefits by mitigating collective stress, promoting psychosocial recovery, and supporting community health.

Moreover, the interplay of distress and resilience observed here mirrors broader models of disaster mental health, which emphasize that economic instability, social fragmentation, and prolonged uncertainty can propagate psychological morbidity across populations [27,28]. Supporting entrepreneurs, therefore, contributes not only to economic restoration but also to the prevention of stress-related disorders and long-term mental health deterioration within affected regions. Integrating mental health promotion and psychosocial support within national recovery frameworks is consistent with WHO’s call for cross-sectoral policies that link economic resilience with population health [24].

## 7. Implications for Policy, Limitations, and Future Work

In practical terms, interventions should prioritize the uncertainty pathway by ensuring clear timelines, predictable aid rules, and rapid communication. In prolonged crises, entrepreneurs’ ability to plan hinges on knowing when and how support will arrive; thus, governments and aid agencies should establish transparent crisis-response protocols that reduce ambiguity. Beyond information provision, programs that cultivate hope and resilience can be integrated into business recovery policies, such as peer support networks, mentoring and coaching schemes, and micro-grants tied to recovery milestones that reinforce a sense of progress. Financial instruments should not only address liquidity gaps but also embed psychological safeguards; for example, staged disbursements linked to capacity-building activities can enhance entrepreneurs’ confidence in their future prospects.

Strengthening institutional trust is equally critical. Transparent, consistent, and reliable delivery mechanisms are essential for restoring procedural certainty and countering perceptions of abandonment. This requires not only efficient implementation of emergency aid but also visible accountability measures by the government, such as independent audits, regular progress reports, and open communication channels with business associations. By embedding clarity and credibility into institutional responses, policymakers can mitigate psychological distress while simultaneously supporting economic resilience. Taken together, these results suggest that MSME policy in wartime should be designed as a joint economic and public mental health intervention. Small business agencies, ministries of finance and economy, and local authorities can use these findings to prioritise uncertainty reduction and transparent procedures in their support schemes, while ministries of health and welfare can embed low-threshold psychological support and peer-based programmes within business assistance initiatives. Integrating these components into existing grant, loan, and tax-relief frameworks may be a cost-effective way to reduce distress, sustain employment, and protect community functioning during future crises.

In interpreting these findings, it is also important to distinguish conceptually between resilience and hope. Whereas resilience reflects a set of cognitive–behavioural coping resources that support effective functioning under adversity, hope represents forward-looking positive expectations about the future. Our results suggest that resilience exerts much of its influence indirectly by sustaining hope, which in turn more proximally shapes emotional responses during acute crisis conditions. Clarifying this distinction helps explain why resilience showed limited direct effects on distress while remaining an important psychological resource in wartime contexts.

Several limitations should be acknowledged. This study is based entirely on self-reported data, which can be affected by a range of biases. Respondents may overstate or understate their levels of distress depending on their current mood, their willingness to disclose difficulties, or the way they interpret survey items [56]. Recall bias is also possible, as participants may have struggled to accurately reconstruct the intensity of their experiences during a highly stressful period [57]. Another limitation concerns the measurement of economic loss. Because we relied on subjective reports rather than verified records, the findings may reflect perceptions of loss rather than actual financial hardship. While perceived economic loss is itself a meaningful driver of distress, the absence of objective data reduces the ability to confirm the reported magnitude of damage.

Additionally, it is important to note that the present study was conducted exclusively among Israeli entrepreneurs who were directly affected by the 7 October attack and the subsequent war. While this focus offers valuable insight into entrepreneurial distress within an acute conflict setting, it also limits the generalizability of the findings. The ongoing regional crisis has resulted in profound psychological, social, and economic consequences for both Israelis and Palestinians. Future research should therefore adopt a broader and more inclusive perspective that examines entrepreneurial distress and resilience among populations exposed to protracted regional conflict. Comparative investigations across diverse sociocultural and geopolitical contexts would enhance external validity and contribute to a more comprehensive understanding of how war-related adversity influences entrepreneurial processes and outcomes.

In addition, the research focuses on a single country during a specific phase of an armed conflict. The conflict began as the country was emerging from COVID-19 and facing political instability, marked by nationwide protests. It opened with a large-scale attack in which Israelis were killed and civilians, including elderly, women, and children, were abducted. This specificity strengthens internal validity but limits generalizability. Comparative or cross-national research is therefore needed to assess whether mechanisms identified here, such as the primacy of uncertainty, the mediating role of hope, and the protective function of institutional trust, hold across different contexts.

These limitations also suggest several avenues for future work. First, because our study relied on self-reported data, subsequent research should incorporate triangulated measures, such as combining survey responses with administrative and financial records, to reduce potential perceptual and recall biases. Second, the focus on a single country during a unique and extreme conflict context highlights the value of cross-national and comparative designs that examine whether the mechanisms identified here (uncertainty as a mediator of revenue loss and hope as a mediator of resilience) can be generalized across different countries. Future within-country research should also incorporate more fine-grained spatial designs that compare entrepreneurs in border-adjacent or heavily exposed areas with those in less directly affected regions, in order to disentangle the effects of structural peripherality from those of heightened proximity to violence.

Moreover, although our sample covered a wide range of sectors, the dispersion of firms across many industries did not provide sufficient statistical power for robust sector by sector comparisons; future research should therefore examine sectoral differences more systematically, for example by oversampling key industries or comparing entrepreneurs in highly exposed sectors (such as tourism and local services) with those embedded in more protected value chains (such as defence related activities).

Third, although our resilience scale captures several core psychological resources, other important dimensions of resilience, such as emotion regulation, meaning-making, and deeper adaptive capacities, were not measured due to the constraints of wartime data collection. Future studies should therefore incorporate broader multidimensional assessments of resilience to better understand its direct and indirect protective effects. Moreover, in our PLS-SEM specification, self-efficacy and perceived social support were modelled as lower-order components of a higher-order resilience construct, so the present analysis captures the combined effect of this resilience-related resource configuration rather than the unique effect of each component. Therefore, future work should compare this higher-order specification with models that treat self-efficacy and social support as separate predictors, in order to disentangle their specific contributions to hope and distress.

Fourth, we did not directly measure respondents’ support for, or identification with, the war itself; future projects should therefore incorporate explicit attitudinal measures of war-related support in order to examine how such positions may moderate the impact of economic loss, uncertainty, and institutional trust on entrepreneurial distress.

Finally, the sample exhibited a substantial gender imbalance, largely due to the wartime context in which many men were unavailable to participate because they were serving in reserve military duty. As a result, gender-related findings should be interpreted with caution, and future research would benefit from samples with greater male representation in order to more accurately assess the role of gender in shaping entrepreneurial distress.

## 8. Conclusions

This study demonstrates that, following a terrorist attack that escalated into a large-scale war, MSME entrepreneurs’ distress stems less from financial loss itself than from the uncertainty it generates. Resilience protects mainly through hope, and trust in government further buffers distress, while women face heightened vulnerability. By modelling resilience hierarchically and pinpointing uncertainty and hope as core mechanisms, the study refines understanding of entrepreneurial well-being under extreme adversity and points to practical levers, policy clarity, reliable support, and resource-building programs that safeguard both entrepreneurs’ mental health and the continuity of their enterprises.

From a public health perspective, these findings highlight that entrepreneurs’ psychological well-being and resilience are integral to community recovery, social stability, and overall population health. Supporting the mental health of small business owners during and after crises not only prevents prolonged distress and economic disruption but also promotes collective resilience and health equity within affected populations. Integrating targeted psychosocial support and resilience-building initiatives into national public health and economic recovery strategies can therefore contribute to sustainable recovery and strengthen societal preparedness for future crises.

## Figures and Tables

**Table 1:** Psychological distress during the war.

Predictor Variable	Coefficient/Test Statistic
Revenue loss	R = 0.113, *p* = 0.03
Resilience	R = −0.127, *p* = 0.01
Trust in government institutions	R = −0.31, *p* < 0.01
Uncertainty	R = 0.456, *p* < 0.01
Hope	R = −0.198, *p* < 0.01
Gender (women > men)	T (df = 361) = −7.05, *p* = 0.01

**Table 2:** Regression model predicting distress during the war.

Variable	B (War)
Revenue Loss	0.003
Resilience	−0.029
Trust in Government	−0.142 ***
Uncertainty About War Duration	0.432 ***
Hope	−0.191 ***
Gender	0.872 ***

Note: *** *p* < 0.01.

## Data Availability

The dataset supporting the findings of this study is openly available in Zenodo at https://doi.org/10.5281/zenodo.17523829, (accessed on 10 December 2025).

## Appendix A. Constructs, Items, Codes, and Sources

**Table:**

Dimension/Construct	Observed/Measured Items	Code	Source
Psychological Distress (DisS_w)	I feel anxiety about the current situation.	Dis1 (q0026_0001)	Adapted from Kessler K6; DASS-21 [25,49]
	I feel stress about the current situation.	Dis2 (q0026_0002)	
	I feel depression about the current situation.	Dis3 (q0026_0003)	
Hope (HOPE_WAR)	I feel that if I work hard, I will find the way for my business to get through the war.	Hope1 (q0026_0006)	Developed for this study, conceptually grounded in Snyder’s hope theory [50] and Herth Hope Index [51]
	I hope that after the war, the future of my business will be good.	Hope2 (q0026_0007)	
	I hope that eventually the war will lead my business to growth.	Hope3 (q0026_0008)	
Trust in Government	I feel that state institutions have supported me as a business owner during the war.	Trust2 (q0025_0002)	Developed for this study, theoretically grounded in Newton and Norris [52]
	The war strengthened my trust in the ability of governmental institutions to cope with emergencies.	Trust3 (q0025_0003)	
	I trust the state and governmental institutions to provide the necessary support to businesses in the future.	Trust4 (q0025_0004)	
	[Reversed item] I do not trust the government.	Trust1R (q0025_0001_R)	
Resilience (RES)	I am looking for creative ways to improve difficult situations.	q0021_0002	Adapted from Connor–Davidson Resilience Scale CD-RISC [53]
	I believe that I can grow in positive ways by coping with difficult situations.	q0021_0003	
	I turn obstacles into positive experiences.	q0022_0011	
	When I face a problem, I take initiative to solve it.	q0022_0013	
	I invest resources in improving work processes in the business.	q0022_0015	
	I am open to continuous learning for self-improvement.	q0022_0016	
	I cope well with situations of uncertainty in my business.	q0022_0024	
	I feel comfortable making decisions even when the available information is incomplete or unclear.	q0022_0025	
Self-Efficacy (SE)	I am good at implementing new ideas and developments.	q0021_0006	Adapted from General Self-Efficacy Scale [54]
	I am the kind of person who "takes matters into my own hands."	q0022_0002	
	I always try to find a way to overcome obstacles; I believe in my ability to overcome challenges.	q0022_0009	
	I believe in my ability to overcome any obstacle.	q0022_0012	
	I am strong at developing new and effective work methods for my business.	q0022_0014	
Self-Efficacy (SE)	I feel high self-confidence in managing my business.	q0022_0018	Adapted from General Self-Efficacy Scale [54]
Uncertainty	Uncertainty regarding the extent of governmental support my business will receive.	UC1 (q0026_0009)	Developed for this study (context-specific, Iron Swords War)
	Uncertainty stemming from the behavior of my customers.	UC2 (q0026_0010)	
	Uncertainty due to not knowing how the war will continue.	UC3 (UN_G)	
Support	My family and community provide me with connections that help me advance my business.	q0019_0003	Developed for this study (context-specific, Iron Swords War)
	Thanks to my family and community support, it is easier for me to cope with business-related stress during the war.	q0019_0004	
	Thanks to my family and community support, it is easier for me to cope with the economic and managerial difficulties that arise during the war.	q0019_0005	
	I received moral support from my family or my community.	q0020_0002	
Support	I received family or community support to cope with personal constraints during the war.	q0020_0003	Developed for this study (context-specific, Iron Swords War)
	My community supported me personally and gave me a sense of belonging and security during the war.	q0020_0006	

## Appendix B. Measurement and Structural Model Results

This appendix reports supplementary analyses from the two-stage PLS-SEM procedure.

Stage 1 (Table A2, Table A3 and Table A4) presents the measurement model assessment, including indicator loadings, reliability, convergent validity, and discriminant validity among the lower-order components of resilience (self-efficacy and support).

Stage 2 (Table A5, Table A6, Table A7, Table A8, Table A9 and Table A10) presents the structural model results, including direct, indirect, and total effects, coefficients of determination (R^2^), collinearity diagnostics (VIF), and discriminant validity among all higher-order constructs.

### Appendix B.1. Measurement Model

**Table A1:** Distribution of businesses by sector *.

N	Sector
7	Industry
19	Finance
17	Real estate, engineering, and renovations
6	Insurance
4	Agriculture
6	Commerce/Trade
19	Tourism
68	Therapy, coaching, guidance, and consulting
29	Software, digital marketing, and management
10	Food (restaurants, patisseries, catering, coffee carts, etc.)
39	Leisure, events, arts, and performance
11	Fashion, clothing, and beauty
10	Writing and translation
5	Health
12	Training, teaching, and education
8	Law and legal services
16	Graphic design, printing, photography, and design
23	Other

* Some businesses were classified into more than one sector.

**Table A2:** Indicator loadings, reliability, and convergent validity.

Construct/Item	Loading (λ)	Cronbach’s α	CR	AVE
Resilience (HOC)		0.886	0.910	0.559
q0021_0002	0.729			
q0021_0003	0.749			
q0022_0011	0.787			
q0022_0013	0.831			
q0022_0015	0.686			
q0022_0016	0.741			
q0022_0024	0.754			
q0022_0025	0.691			
Self-Efficacy		0.884	0.912	0.634
q0021_0006	0.776			
q0022_0002	0.807			
q0022_0009	0.852			
q0022_0012	0.797			
q0022_0014	0.797			
q0022_0018	0.746			
Support		0.855	0.893	0.584
q0019_0003	0.660			
q0019_0004	0.848			
q0019_0005	0.849			
q0020_0002	0.776			
q0020_0003	0.715			
q0020_0006	0.719			
Hope		0.780	0.872	0.694
q0026_0006	0.797			
q0026_0007	0.850			
q0026_0008	0.850			
Trust		0.836	0.882	0.653
q0025_0001_R	0.833			
q0025_0002	0.707			
q0025_0003	0.809			
q0025_0004	0.874			
Uncertainty		0.624	0.882	0.653
UN_G	0.857			
q0026_0000	0.695			
q0026_0010	0.693			
Distress		0.881	0.927	0.81
DIS3	0.818			
q0026_0001	0.941			
q0026_0002	0.935			

Note: Loading (λ) = strength of the relationship between an indicator and its latent construct; Cronbach’s α = internal consistency reliability; CR = composite reliability; AVE = average variance extracted. Common thresholds are λ > 0.70, α ≥ 0.70, CR ≥ 0.70, and AVE ≥ 0.50.

**Table A3:** Discriminant validity: heterotrait–monotrait ratio (HTMT).

	Self-Efficacy
Support	0.447

Note: HTMT = heterotrait–monotrait ratio of correlations. Values below 0.85 indicate satisfactory discriminant validity [58].

**Table A4:** Discriminant validity: Fornell–Larcker criterion.

	Self-Efficacy	Support
Self-Efficacy	0.796	
Support	0.39	0.764

Note. Diagonal values are the square root of the average variance extracted (AVE). For discriminant validity, each diagonal value should exceed the corresponding off-diagonal correlations [59].

### Appendix B.2. Structural Model

**Table A5:** Path coefficients (direct effects).

	Original Sample (O)	Sample Mean (M)	Standard Deviation (STDEV)	T Statistics	*p* Values
Gender -> Distress	0.701	0.705	0.112	6.278	<0.001
Hope -> Distress	−0.151	−0.151	0.046	3.3	0.001
Resilience (HOC) -> Distress	−0.024	−0.024	0.049	0.496	0.62
Resilience (HOC) -> Hope	0.378	0.383	0.047	8.05	<0.001
Revenue Loss -> Distress	−0.006	−0.007	0.043	0.149	0.882
Revenue Loss -> Uncertainty	0.232	0.233	0.048	4.844	<0.001
Trust in Gov -> Distress	−0.158	−0.162	0.052	3.061	0.002
Uncertainty -> Distress	0.358	0.359	0.054	6.635	<0.001

**Table A6:** Specific indirect effects.

	Original Sample (O)	Sample Mean (M)	Standard Deviation (STDEV)	T Statistics	*p* Values
Resilience (HOC) -> Hope -> Distress	−0.057	−0.057	0.019	3.074	0.002
Revenue Loss -> Uncertainty -> Distress	0.083	0.083	0.021	3.989	<0.001

**Table A7:** Total effects.

	Original Sample (O)	Sample Mean (M)	Standard Deviation (STDEV)	T Statistics	*p* Values
Gender -> Distress	0.701	0.705	0.112	6.278	<0.001
Hope -> Distress	−0.151	−0.151	0.046	3.3	0.001
Resilience (HOC) -> Distress	−0.081	−0.081	0.045	1.798	0.072
Resilience (HOC) -> Hope	0.378	0.383	0.047	8.05	<0.001
Revenue Loss -> Distress	0.077	0.077	0.047	1.628	0.104
Revenue Loss -> Uncertainty	0.232	0.233	0.048	4.844	<0.001
Trust in Gov -> Distress	−0.158	−0.162	0.052	3.061	0.002
Uncertainty -> Distress	0.358	0.359	0.054	6.635	<0.001

**Table A8:** Coefficient of determination (R^2^).

	Original Sample (O)	Sample Mean (M)	Standard Deviation (STDEV)	T Statistics	*p* Values
Distress	0.361	0.377	0.042	8.595	<0.001

**Table A9:** Collinearity assessment (VIF values).

	Original Sample (O)
Gender -> Distress	1.023
Hope -> Distress	1.215
Resilience (HOC) -> Distress	1.177
Resilience (HOC) -> Hope	1
Revenue Loss -> Distress	1.089
Revenue Loss -> Uncertainty	1
Trust in Gov -> Distress	1.201
Uncertainty -> Distress	1.222

**Table A10:** Discriminant validity (HTMT ratios).

	Distress	Gender	Hope	Revenue Loss	Trust in Gov	Uncertainty
Distress						
Gender	0.375					
Hope	0.23	0.072				
Revenue Loss	0.123	0.033	0.173			
Trust in Gov	0.35	0.076	0.237	0.159		
Uncertainty	0.591	0.149	0.112	0.293	0.435	
Resilience (HOC)	0.145	0.027	0.468	0.144	0.153	0.148
